# Body mass index trajectories and mortality risk in Japan using a population-based prospective cohort study: the Japan Public Health Center-based Prospective Study

**DOI:** 10.1093/ije/dyad145

**Published:** 2023-10-25

**Authors:** Nao Yamamoto, Keisuke Ejima, Luis M Mestre, Arthur H Owora, Manami Inoue, Shoichiro Tsugane, Norie Sawada

**Affiliations:** School of Human Evolution and Social Change, Arizona State University, Tempe, AZ, USA; Lee Kong Chian School of Medicine, Nanyang Technological University, Singapore, Singapore; Department of Global Health Policy, Graduate School of Medicine, The University of Tokyo, Tokyo, Japan; Department of Psychiatry, Yale University, New Haven, CT, USA; Department of Epidemiology and Biostatistics, Indiana University School of Public Health-Bloomington, Bloomington, IN, USA; Department of Pediatrics, Indiana University School of Medicine-Indianapolis, Indianapolis, IN, USA; Division of Cohort Research, National Cancer Center Institute for Cancer Control, Tokyo, Japan; Division of Prevention, National Cancer Center Institute for Cancer Control, Tokyo, Japan; Division of Cohort Research, National Cancer Center Institute for Cancer Control, Tokyo, Japan; Division of Cohort Research, National Cancer Center Institute for Cancer Control, Tokyo, Japan

**Keywords:** Japan Public Health Center-based Prospective Study, latent class growth model, body mass index trajectory

## Abstract

**Background:**

Recent studies have found that long-term changes in weight during adulthood are associated with a high risk of mortality. The objective of this study was to characterize body mass index (BMI) trajectories during adulthood and to examine the association between BMI trajectories and risk of death in the Japanese population.

**Methods:**

The data were extracted from Japan Public Health Center-based Prospective Study—a population-based prospective cohort study in Japan with participants aged 40–69 years followed over 20 years. The participants were categorized into multiple BMI trajectory groups using the latent class growth model. The Cox proportional-hazards model was conducted using all-cause mortality and cause-specific mortality as outcomes and the identified BMI trajectory groups as a predictor. In total, 65 520 participants were included in the analysis.

**Results:**

Six BMI trajectory groups were identified: underweight stable (Group 1), low-to-high normal (Group 2), high-to-low normal (Group 3), normal to overweight (Group 4), overweight to normal (Group 5) and normal to obese (Group 6). Our Cox models showed a higher hazard (risk) of all-cause mortality among participants in the BMI-declining groups [Group 3, adjusted hazard ratio (aHR): 1.10, 95% CI: 1.05–1.16; Group 5, aHR: 1.16, 95% CI: 1.08–1.26], underweight stable group (Group 1, aHR: 1.27, 95% CI: 1.21–1.33) and normal to obese group (Group 6, aHR: 1.22, 95% CI: 1.13–1.33) than Group 2 (low-to-high normal BMI trajectory).

**Conclusions:**

Stable underweight and weight loss were associated with a high risk of mortality, both of which were uniquely observed in a Japanese population.

Key Messages

- Obesity is known to be associated with an increased risk of chronic diseases and mortality; however, it is unclear whether long-term weight loss or body mass index (BMI) at any single time point during adulthood explains the excess mortality risk.
- Our study objective was to characterize BMI trajectories during adulthood (after age 20 years) and to examine the association between identified BMI trajectories and risk of death in the Japanese population.
- We identified six groups with unique BMI trajectories in a Japanese cohort.
- Stable underweight and weight-loss trajectory groups were associated with a higher risk of mortality than overweight trajectory groups.
- Although a single time-point BMI is still useful, monitoring BMI over time may be more critical to determining mortality risk.

## Introduction

Obesity is on an upward trend worldwide, regardless of age, sex or social status.^1^ Due to its association with numerous conditions predisposing to life-threatening diseases (e.g. cardiovascular diseases, type 2 diabetes, liver disease and several types of cancers), treating people with obesity is considered crucial for improving both quality of life and life expectancy.^2^ Indeed, obesity has been globally recognized as a chronic disease; thus, health practitioners are encouraged to monitor individuals’ weight status and treat obesity through behavioural, pharmacological and surgical interventions. These interventions have been shown to effectively reduce the risk of obesity-related diseases.^3^^,^^4^ In addition, underweight is associated with risk of mortality regardless of ethnicity.^5–7^ However, although a high body mass index (BMI) was associated with a high mortality risk in many countries/regions, this association is not observed in South Asia.^5^^,^^7^

BMI plays a central role in assessing obesity, especially in epidemiological studies, due to its strong correlation with body fat percentage at the population level, making it a surrogate measure for adiposity.^8^ Furthermore, measuring BMI (height and weight) is simple and convenient for use in epidemiological surveillance, especially when targeting large populations. This is because conducting detailed assessments of medical and physical conditions, which can be costly and laborious, might not always be feasible. In most epidemiological studies, a single time-point BMI has been used to assess the risk of obesity-related diseases and mortality. However, a meta-analysis reported that both weight loss and weight gain in midlife are associated with high all-cause and cardiovascular disease mortality risks.^9^ Recent studies have also investigated the utility of past BMI in addition to BMI at a given time point in assessing the risk of diseases and mortality. For instance, Stokes found that mortality risk differs based on a maximum lifetime BMI among individuals in the same BMI category at the time of the survey (in an older cohort), particularly noting higher mortality risk among those who experienced weight loss,^10^^,^^11^ possibly due to underlying diseases. Zheng's research suggests that the effect of weight gain on mortality is influenced by an individual’s BMI trajectory over time.^12^^,^^13^ A recent study investigated the BMI trajectory in a Japanese cohort prior to the onset of type 2 diabetes.^14^ Some studies identified the BMI trajectory over age (i.e. age as timescale) from childhood to early adulthood,^15–17^ revealing associations with hypertension risk in the Chinese population^16^ and all-cause and cause-specific mortality in the Australian population.^17^ Other studies focusing on late adulthood did not observe the identification of a weight-gain group in Korean and Japanese cohorts and, even when identified, the association between weight gain and mortality risk was not demonstrated.^18–20^

However, the pattern of lifetime BMI trajectory during adulthood, encompassing both early and late adulthood in the Japanese population and its association with the risk of obesity-related diseases and mortality, remains unclear. In this study, we analysed longitudinal BMI data retrieved from the Japan Public Health Center-based Prospective Study (JPHC). First, we categorized the JPHC participants aged 40–69 years into several groups using the latent class growth model to identify typical BMI patterns. Second, we computed the all-cause and cause-specific mortality risk of those identified groups using the Cox proportional-hazards model. The objective of this study was to analyse the BMI trajectory to assess mortality risk in the Japanese population. Based on previous studies emphasizing the importance of continuous BMI monitoring over time rather than relying on a single time point, we hypothesized that certain BMI trajectory groups would become distinguishable at specific ages as exhibiting varying mortality risks.

## Methods

### Study design and data

We used data from a population-based prospective cohort study in Japan—the JPHC, which is a study designed to investigate lifestyle-related risk factors for diseases, including lifestyle habits.^21^ The participants aged 40–69 years were followed over 20 years. The details of the data are described in the Supplementary Materials (available as Supplementary data at *IJE* online).

### Classification of longitudinal BMI data using latent class growth model and survival analysis

The longitudinal BMI data were classified into multiple groups using a latent class growth model with linear, quadratic or cubic functions of age (in years) for class-specific effects (i.e. a fixed effect is not shared among the groups).^22^ We assumed that the class-specific random effects were independent of age and the residual variances were invariant across latent classes. No covariates were included in the class-membership model. To determine the best-fitting model among those with one to eight trajectories, we followed the recommendations provided by Nagin^23^ and used the Akaike information criterion (AIC), Bayesian information criterion (BIC) and entropy measures, along with our content knowledge. We also examined the 95% confidence intervals of emergent trajectory groups to rule out group overlap. The content knowledge was based on previous research^17^ and reference to BMI categories was defined by the World Health Organization.^24^ Participants were assigned to the group with the highest posterior class-membership probability. We adhered to the GRoLTS Checklist Guidelines to report the latent class growth model.^25^

Subsequently, we performed survival analysis using the Cox proportional-hazards model with age as the timescale in which the BMI trajectory groups were used as a predictor and all-cause and cause-specific mortality as an outcome. The model was adjusted for sex, cigarette smoking (never smokers, former smokers, current smokers), alcohol consumption (non-drinkers, occasional drinkers, 1–149, 150–299, 300–449, >450 g/week), time engaged in sports and physical exercise in free time [limited (< once/month), 1–3 times/month, 1–2 times/week, 3–4 times/week, every day], history of diabetes and history of hypertension, assessed at baseline and stratified by the public health centres. The same analyses were performed for cause-specific mortalities {i.e. cancer [the 10th revision of the International Statistical Classification of Diseases and Related Health Problems (ICD10): C00 to C97], heart disease [ICD10: I20 to I52], cerebrovascular diseases [ICD10: I60 to I69], respiratory diseases [ICD10: J10 to J18 and J40 to J47] and others}. The assumption of the Cox proportional hazard model was tested using Schoenfeld residuals.^26^ The analysis was stratified by the covariates significantly violating the proportionality assumption. Individuals with missing data were excluded from the survival analysis. Subgroup analyses were conducted separately for male and female participants and for participants without any history of diseases that could cause weight change (cerebrovascular disease, ischaemic heart disease and cancer) at baseline.

We performed all the simulations using the statistical computing software R 4.0.1 (R Development Core Team). The R package ‘lcmm’ was used to implement the latent class growth model.

### Patient and public involvement

No patients were involved in designing the study, implementing the study findings, setting study questions or setting the study outcomes directly. All the participants of the JPHC are community residents. We have been regularly organizing meetings with health practitioners in the study areas to discuss health practice since we formed this study cohort.

## Results

**Figure 1.**
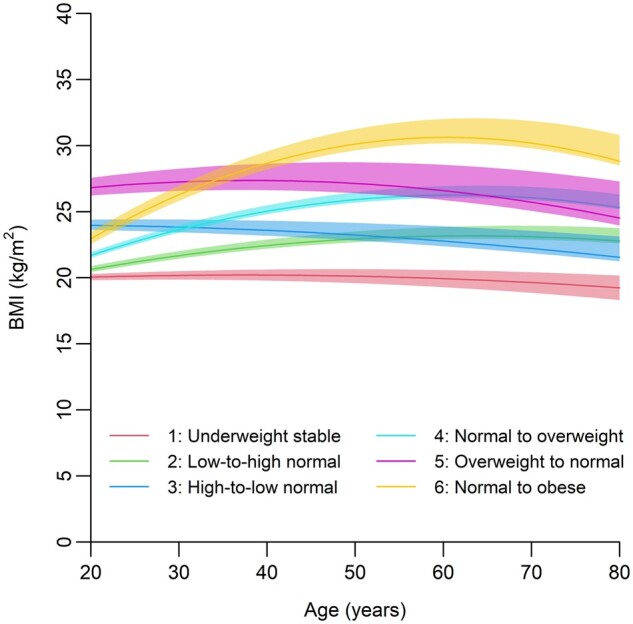
Body mass index change trajectories of six groups. Each line and shaded area represent the expected weight trajectory of each group, with the 95% confidence interval

A total of 65 520 participants were included in the analysis (Supplementary Figure S1, available as Supplementary data at *IJE* online). Quadratic functions were used to model the BMI trajectories, which resulted in lower AIC and BIC values compared with linear and cubic models (Supplementary Table S1, available as Supplementary data at *IJE* online). The latent class trajectory analysis revealed that increasing the number of identified BMI groups led to improved BIC values (Supplementary Table S2, available as Supplementary data at *IJE* online). The order of entropy, indicating the quality of classification, ranked as follows: one-group model, four-group model, three-group model, six-group model and seven-group model. Considering the balance between simplicity and reliability, a six-group model was selected for further analysis. Table 1 shows the trajectories of BMI and other variables based on the summary statistics derived from this model. The identified six BMI trajectory groups (Figure 1 and Table 1) were named based on the previous study adapting WHO’s classifications:^17^ underweight stable (Group 1), low-to-high normal (Group 2), high-to-low normal (Group 3), normal to overweight (Group 4), overweight to normal (Group 5) and normal to obese (Group 6). Until the age of 60 years, three groups experienced BMI gain, whereas two groups experienced BMI loss. The three groups with continuous BMI gain reached their peak around the age of 60 years and then their BMI started to decline or remain stable. Regardless of the dynamic BMI changes, the trend of four groups, except for the BMI loss groups (Groups 3 and 5), remained consistent over time. Group 5 (BMI loss group) started as overweight at the age of 20 years, the highest among the six groups, and gradually transitioned to normal weight, becoming indistinguishable from Group 4, which presented BMI gain after the age of 50 years. Similarly, the BMI at the age of 20 years in Group 3 was higher than that of Group 2, but the difference had diminished by the age of 50 years.

**Table 1.** Summary statistics of the participants^a^

Characteristic	Group 1 (underweight stable)	Group 2 (low-to-high normal)	Group 3 (high-to-low normal)	Group 4 (normal to overweight)	Group 5 (overweight to normal)	Group 6 (normal to obese)
Total number	13 800	22 694	7742	15 797	2481	3006
Length of follow-up (years)	23.8 (3.9)	24.4 (3.5)	23.8 (4.1)	24.6 (3.4)	23.9 (3.8)	24.4 (3.6)
Person-years	328 268.3	553 266.1	184 441.7	388 049.8	59 343.8	73 361.3
Proportion of groups (%)	21.1	34.6	11.8	24.1	3.8	4.6
Proportion of men (%)	40.3	46.2	49.2	48.1	51.4	38.8
Age at baseline (years)	50.5 (20)	50.5 (22.8)	54.1 (23)	50.7 (25.8)	54.2 (26.8)	50.7 (29.9)
BMI (kg/m^2^)	20 (1.3)	22.8 (1.3)	23 (1.4)	25.8 (1.4)	26.8 (1.8)	29.9 (2.1)
Alcohol consumption (%)						
Non-drinkers	53.7	50.0	51.7	50.5	53.1	59.5
Occasional drinkers	9.6	10.2	8.6	11.2	9.8	11.3
1–149 g/week	16.7	17.0	14.7	15.3	13.0	12.1
150–299 g/week	9.7	11.1	11.0	11.0	10.7	7.1
300–449 g/week	5.7	6.9	7.2	6.3	7.0	4.6
>450 g/week	4.6	4.8	6.8	5.7	6.3	5.4
Cigarette smoking (%)						
<1 day/month	62.2	61.8	57.6	62.6	61.1	68.1
1–3 days/month	8.7	13.0	12.4	13.9	13.0	11.3
>1 day/week	29.1	25.2	30.0	23.4	25.9	20.6
Sports and physical exercise in leisure time (%)						
Limited (< once/month)	71.7	66.6	72.1	67.3	70.1	69.4
1–3 times/month	10.6	13.5	11.0	12.6	11.0	10.9
1–2 times/week	9.6	10.6	7.8	10.3	8.0	9.9
3–4 times/week	3.8	4.6	4.0	4.8	5.2	4.3
Every day	4.2	4.6	5.0	4.9	5.7	5.6
Diabetes (%)	3.2	3.5	6.1	4.1	7.0	5.2
Hypertension (%)	9.0	14.1	16.8	20.5	25.7	28.3
Medical history^b^ (%)	3.1	2.9	3.9	3.1	4.7	3.7

BMI, body mass index.

a All information is at the baseline survey. Means and standard deviations (in parentheses) are presented for continuous variables. Proportions within the groups are presented for categorical variables except ‘Proportion of groups’.

b Those who reported any of the following medical history: cerebrovascular disease, ischaemic heart disease and cancer.

Table 1 and Supplementary Table S3 (available as Supplementary data at *IJE* online) summarize the characteristics of the six BMI trajectory groups. The largest group was Group 2 (34.6%), in which BMI increased but stayed within the normal range (20.0–25.0 kg/m^2^) over time. The BMI loss groups (Groups 3 and 5) accounted for only 15.6% in total. Group 1 (underweight stable) and Group 6 (normal to obese) were composed of more women, whereas the BMI loss groups (Groups 3 and 5) were composed of more men compared with the other groups. A clear difference between the groups was not observed in behavioural variables (alcohol consumption, cigarette smoking and sports and physical exercise). The BMI loss groups (Groups 3 and 5) presented a higher prevalence of diabetes and other medical history, which may suggest that some participants in those groups lost weight due to those health conditions. The prevalence of hypertension was correlated with the baseline BMI.

The Cox proportional-hazards model was used to assess the difference in mortality between the BMI trajectory groups. Group 2 (low-to-high normal) was selected as the reference group for two reasons. First, Group 2 consisted of individuals with a BMI within the normal range, allowing a comparison of mortality risks between underweight (Group 1) and overweight/obesity (Group 3, 4 and 5) relative to this baseline. Second, Group 2 represented the largest group within the study population. Because sex and smoking status violated the proportionality assumption, the model was stratified by those two variables. When the whole sample was analysed, all-cause mortality risk among all groups except Group 4 was significantly higher than that of Group 2. This finding suggests that losing weight [Group 3, adjusted hazard ratio (aHR): 1.10, 95% CI: 1.05–1.16; Group 5, aHR: 1.16, 95% CI: 1.08–1.25] and maintaining underweight (Group 1, aHR: 1.26, 95% CI: 1.21–1.32) are associated with high mortality (Table 2). Gaining weight is not necessarily associated with high mortality risk. However, the risk was significantly higher in Group 6 (aHR: 1.22, 95% CI: 1.13–1.33), which consistently presented the highest BMI after the age of 50 years. A similar trend was observed for cause-specific mortalities. The high mortality risk of Group 1 was emphasized by deaths due to respiratory diseases (aHR: 2.30, 95% CI: 1.94–2.72), suggesting that maintaining underweight was strongly associated with a high mortality risk due to respiratory diseases. We confirmed that most results for the whole sample were consistent with those for the male and female participants. We observed the same trend when the analysis was focused on those without medical history, which is a potential confounder of the association between weight change and mortality, suggesting that weight change may have a causal effect on mortality risk.

**Table 2.** Adjusted hazard ratios of all-cause and cause-specific mortality of different body mass index trajectory groups

	Group 1 (underweight stable)			Group 2 (low-to-high normal)			Group 3 (high-to-low normal)			Group 4 (normal to overweight)			Group 5 (overweight to normal)			Group 6 (normal to obese)		
Both sexes^a^	Risk pop	Death	aHR (95% CI)	Risk pop	Death	aHR	Risk pop	Death	aHR (95% CI)	Risk pop	Death	aHR (95% CI)	Risk pop	Death	aHR (95% CI)	Risk pop	Death	aHR (95% CI)
All-cause	13 800	3120	1.26 (1.21–1.32)*	22 694	4176	1	7742	2446	1.10 (1.05–1.16)*	15 797	2935	0.99 (0.94–1.04)	2481	805	1.16 (1.08–1.25)*	3006	645	1.22 (1.13–1.33)*
Cancer		1009	1.14 (1.05–1.24)*		1469	1		737	1.01 (0.92–1.10)		1024	1.00 (0.93–1.09)		253	1.15 (1.01–1.32)*		221	1.29 (1.11–1.48)*
Heart		334	1.24 (1.08–1.43)*		472	1		333	1.33 (1.16–1.54)*		362	1.04 (0.91–1.20)		112	1.34 (1.08–1.65)*		97	1.47 (1.18–1.84)*
CeVD		262	1.27 (1.08–1.49)*		360	1		225	1.18 (0.99–1.39)		248	0.94 (0.80–1.10)		83	1.34 (1.05–1.71)*		46	0.98 (0.72–1.34)
Respiratory disease		332	2.30 (1.94–2.72)*		249	1		226	1.53 (1.27–1.84)*		160	0.89 (0.73–1.09)		42	0.92 (0.66–1.28)		28	0.87 (0.59–1.30)
Other		1185	1.23 (1.14–1.32)*		1626	1		925	1.04 (0.96–1.13)		1141	0.99 (0.92–1.07)		316	1.13 (1.00–1.27)		253	1.21 (1.05–1.38)*

**Male** ^b^	**Risk pop**	**Death**	**HR (95% CI)**	**Risk pop**	**Death**	**HR**	**Risk pop**	**Death**	**HR (95% CI)**	**Risk Pop**	**Death**	**HR (95% CI)**	**Risk pop**	**Death**	**HR (95% CI)**	**Risk pop**	**Death**	**HR (95% CI)**

All-cause	5567	1916	1.28 (1.21–1.36)*	10 489	2597	1	3806	1620	1.13 (1.06–1.20)*	7599	1737	0.99 (0.93–1.06)	1275	477	1.20 (1.09–1.33)*	1165	283	1.25 (1.10–1.42)*
Cancer		656	1.21 (1.09–1.33)*		934	1		504	1.02 (0.92–1.14)		639	1.02 (0.92–1.13)		162	1.19 (1.00–1.41)*		107	1.32 (1.08–1.62)*
Heart		188	1.13 (0.94–1.35)		302	1		203	1.24 (1.03–1.48)*		217	1.06 (0.88–1.26)		66	1.39 (1.06–1.82)*		41	1.47 (1.06–2.05)*
CeVD		155	1.32 (1.07–1.63)*		210	1		154	1.32 (1.07–1.63)*		145	0.99 (0.80–1.23)		50	1.51 (1.10–2.06)*		21	1.15 (0.73–1.82)
Respiratory disease		240	2.43 (1.99–2.96)*		172	1		180	1.69 (1.37–2.09)*		105	0.89 (0.69–1.13)		27	0.94 (0.62–1.41)		18	1.13 (0.69–1.84)
Other		679	1.21 (1.09–1.33)*		979	1		579	1.05 (0.95–1.17)		631	0.97 (0.88–1.07)		173	1.15 (0.98–1.36)		96	1.16 (0.94–1.43)

**Female** ^b^	**Risk pop**	**Death**	**HR (95% CI)**	**Risk pop**	**Death**	**HR**	**Risk pop**	**Death**	**HR (95% CI)**	**Risk pop**	**Death**	**HR (95% CI)**	**Risk pop**	**Death**	**HR (95% CI)**	**Risk pop**	**Death**	**HR (95% CI)**

All-cause	8233	1204	1.23 (1.14–1.33)*	12 205	1579	1	3936	826	1.07 (0.98–1.16)	8198	1198	0.98 (0.91–1.06)	1206	328	1.10 (0.97–1.24)	1841	362	1.17 (1.04–1.32)*
Cancer		353	1.04 (0.91–1.20)		535	1		233	0.98 (0.84–1.15)		385	0.98 (0.86–1.12)		91	1.08 (0.87–1.36)		114	1.23 (1.00–1.50)
Heart		146	1.44 (1.15–1.81)*		170	1		130	1.54 (1.22–1.94)*		145	1.03 (0.82–1.28)		46	1.28 (0.92–1.78)		56	1.44 (1.06–1.96)*
CeVD		107	1.18 (0.92–1.52)		150	1		71	0.95 (0.72–1.27)		103	0.85 (0.66–1.10)		33	1.12 (0.76–1.64)		25	0.83 (0.54–1.27)
Respiratory disease		92	1.98 (1.45–2.69)*		77	1		46	1.13 (0.78–1.64)		55	0.88 (0.62–1.24)		15	0.86 (0.49–1.51)		10	0.58 (0.30–1.14)
Other		506	1.25 (1.11–1.41)*		647	1		346	1.04 (0.91–1.18)		510	1.02 (0.91–1.15)		143	1.09 (0.91–1.31)		157	1.22 (1.02–1.45)*

**Without medical history** ^c^	**Risk pop**	**Death**	**HR (95% CI)**	**Risk pop**	**Death**	**HR**	**Risk pop**	**Death**	**HR (95% CI)**	**Risk pop**	**Death**	**HR (95% CI)**	**Risk pop**	**Death**	**HR (95% CI)**	**Risk pop**	**Death**	**HR (95% CI)**

All-cause	13 371	2946	1.26 (1.20–1.32)*	22 032	3942	1	7442	2311	1.11 (1.06–1.17)*	15 308	2761	1.01 (0.96–1.06)	2365	748	1.18 (1.09–1.27)*	2895	613	1.30 (1.19–1.42)*
Cancer		964	1.16 (1.06–1.25)*		1398	1		709	1.02 (0.93–1.12)		969	1.00 (0.92–1.09)		238	1.15 (1.00–1.33)*		212	1.31 (1.14–1.52)*
Heart		309	1.21 (1.04–1.40)*		436	1		305	1.33 (1.15–1.54)*		335	1.09 (0.94–1.26)		105	1.43 (1.15–1.77)*		89	1.64 (1.30–2.06)*
CeVD		239	1.21 (1.02–1.43)*		334	1		208	1.18 (0.99–1.41)		225	0.96 (0.81–1.14)		71	1.33 (1.02–1.72)*		45	1.16 (0.85–1.59)
Respiratory disease		318	2.27 (1.91–2.69)*		238	1		206	1.46 (1.21–1.77)*		153	0.92 (0.75–1.12)		37	0.88 (0.62–1.25)		25	0.87 (0.57–1.32)
Other		1118	1.22 (1.13–1.32)*		1536	1		883	1.06 (0.98–1.15)		1079	1.01 (0.93–1.09)		298	1.15 (1.02–1.31)*		242	1.28 (1.12–1.47)*

aHR, adjusted hazard ratio; CeVD, cerebrovascular disease; HR, hazard ratio.

a Adjusted by alcohol consumption (non-drinkers, occasional drinkers, 1–149 g/week, 150–299 g/week, 300–449 g/week, >450 g/week), time engaged in sports and physical exercise in free time (<1 day/month, 1–3 days/month, >1 day/week), history of diabetes and history of hypertension assessed at baseline and stratified by the JPHC communities, cigarette smoking (never smokers, past smokers, current smokers) and sex.

b Adjusted and stratified by all variables in ^a^ except sex.

c Only those without medical history (cerebrovascular disease, ischaemic heart disease and cancer) at baseline are included in the analysis. Adjusted and stratified by all variables in ^a^ except diabetes and hypertension.

* *P*-value < 0.05.

## Discussion

We analysed the longitudinal BMI data of the Japanese population using a latent class growth model. Six BMI trajectory groups were identified: underweight stable (Group 1), low-to-high normal (Group 2), high-to-low normal (Group 3), normal to overweight (Group 4), overweight to normal (Group 5) and normal to obese (Group 6). When using Group 2 as the reference group, all groups except Group 4 exhibited hazard ratios for all-cause mortality that were significantly larger than 1.

There appears to be a difference in BMI trajectories and mortality risk between Western and Asian populations. In an Australian cohort study, no weight-loss or underweight groups were identified,^17^ both of which were identified and associated with high mortality risk in our study.

We are not the first to investigate the association between BMI trajectory and mortality risk in Asian populations. Previous studies analysed data of Korean,^18^ Japanese^19^ and Chinese cohorts.^27^ The overall findings of these studies align with our own, indicating that weight loss is prevalent and associated with a higher mortality risk in Asian populations.

Weight gain during young to middle adulthood has been associated with a higher mortality risk.^28^ However, when focusing on late adulthood, the weight-gain group was not identified in the Korean cohort^18^ and the Japanese cohort.^19^ Even in cases in which a weight-gain group was identified, the mortality risk was not significantly different from that of the stable-weight group in the Japanese cohort.^20^ Although the Chinese cohort (aged ≥45 years) did observe a slightly higher mortality risk in the weight-gain group, this significance disappeared in a sensitivity analysis focusing on individuals aged ≥60 years.^27^ In our study, Group 6 (normal to obese) experienced weight gain by the age of 50 years, after which their weight remained stable. This group presented a high mortality risk, consistently with the previous findings.

The association between weight loss and high mortality should be carefully interpreted in terms of causation. Previous studies have suggested that intentional weight loss without medical conditions may be associated with lower mortality risk, suggesting a potential benefit of weight loss. However, the causal effect of weight loss on mortality risk cannot be assessed. Unintentional weight loss, often associated with underlying medical conditions, is a known risk factor for higher mortality and medical conditions could confound the association between BMI change and health outcomes.^29–32^ In our study, the weight-loss groups had a high prevalence of diabetes and other medical history. We lack information on the intentionality of weight loss, highlighting the need for further investigation into its impact on mortality risk in the Japanese population. We performed an analysis focusing on individuals without any medical history to minimize its influence but weight loss remained associated with higher mortality, suggesting a potential causal effect of weight loss on mortality that is not solely due to confounding by medical history. Careful consideration is encouraged in the interpretation, as some diseases might not be diagnosed or reported.

The utility of BMI in epidemiological studies is well known but the limitations in clinical settings have also been discussed. One of the key limitations is that BMI cannot accurately predict adiposity at the individual level, as it does not differentiate between fat mass and fat-free mass.^33^^,^^34^ Additionally, the distribution of BMI varies across age, sex and racial/ethnic groups. For example, individuals classified as non-obese based on BMI may still have a substantially higher risk of type 2 diabetes in the Asian population,^35^ highlighting the need for customized BMI cut-offs for obesity, as the standard cut-off (<30 kg/m^2^) may not be universally applicable.^36^^,^^37^ To overcome the clinical limitations of BMI, multiple measurements are comprehensively considered in obesity-related risk assessments, including medical and physical conditions (BMI, waist circumference, blood cholesterol level, blood pressure and blood glucose level).^38^ Several obesity staging systems, such as the Edmonton Obesity Staging System (EOSS)^39^ and Cardiometabolic Disease Staging (CMDS), integrate these measurements to categorize individuals based on overall cardiometabolic conditions.^34^^,^^40^ Further studies are needed to investigate the association between BMI trajectory and other measurements of medical and physical conditions, providing a comprehensive understanding of the effects of the BMI trajectory on disease onset and progression, and enabling more accurate assessments of disease and mortality risks.

There are several strengths of this study. The first and main strength of our study is that we classified BMI trajectories over the entire adulthood period (20–69 years) in the Japanese population. Unlike previous studies that often focused on specific age ranges and used simpler approaches to characterize BMI/weight changes (e.g. using a cut-off of >5 kg for weight gain),^41–46^ our study provides a comprehensive and detailed analysis of long-term weight trajectories. Second, most previous studies used follow-up time or time before specific events as the timescale (i.e. not age as a timescale) for classifying the BMI trajectories, which typically resulted in relatively short follow-up durations (<10 years).^19^^,^^27^ In contrast, our study employed age as the timescale, allowing the capture of weight changes from early adulthood to late age, offering a more robust and comprehensive understanding of BMI trajectories throughout the lifespan. Third, the data from JPHC include those for diverse participants from public health centres in prefectures, enhancing the generalizability of the findings to the broader Japanese population. However, further validation studies are warranted to confirm the applicability of our results.

Several limitations of this study should be noted. First, the BMI data used are self-reported and there are no validation data available specifically for self-reported BMI data at age 20 years. Although self-reported BMI is known to induce attenuation bias in assessing the association between BMI and mortality in general, the measurement bias in the JPHC survey was mild compared with those of other relevant studies. Self-reported BMI was only slightly lower (0.1 kg/m^2^ for both sexes) than measured BMI.^47^ Moreover, a high Spearman’s correlation coefficient between BMI collected at health check-ups and self-reported BMI (0.91 in men and 0.92 in women) was observed, as confirmed in selected participants at the baseline survey.^47^ Second, the analysis was performed using the BMI data from four different time points; however, we are uncertain whether four time points are sufficient to capture the BMI trajectory during adulthood. Third, individuals with missing data were excluded from the survival analysis, which might have induced selection biases as previously discussed.^48^^,^^49^ If necessary, multiple imputation or other approaches to treat missingness would be advisable.^50^ Fourth, unmeasured confounding or time-varying confounders were not considered in this study, which may have affected our results. Additionally, we may also need to consider the direction of the biases, as discussed in Yland *et al.*^51^

Stable underweight and weight loss were associated with a high risk of mortality, both of which have been uniquely observed in Asian populations, including the Japanese population. BMI has been used in both epidemiological and clinical studies as an important predictor of obesity-related disease and mortality risk. Although a single time-point BMI is still useful, our study emphasized the importance of monitoring BMI over time. The longitudinal BMI data helped identify a population with a higher mortality risk, despite similar BMI at specific ages.

## Ethics approval

IRB review was exempted at Indiana University (#2001615938), National Cancer Center (IRB-2015-085), and Nanyang Technological University (IRB-2023–095).

## Supplementary Material

dyad145_Supplementary_Data

## Data Availability

For information on how to submit an application for gaining access to JPHC data and/or biospecimens, please follow the instructions at https://epi.ncc.go.jp/en/jphc/805/8155.html.
