# Supplementary material for: Body mass index trajectories and mortality risk in Japan using a population-based prospective cohort study: the Japan Public Health Center-based Prospective Study
Source: Int J Epidemiol. 2023 Oct 25;53(1):dyad145. doi: 10.1093/ije/dyad145 (PMC10859135; doi:10.1093/ije/dyad145)
Supplement: dyad145_Supplementary_Data [file dyad145_supplementary_data.docx]

**Supplementary Materials**

**Study design and data**

We used data from a population-based prospective cohort study in Japan: Japan Public Health Center-based Prospective Study (JPHC), a study designed to investigate lifestyle-related risk factors for diseases including lifestyle habits^1^. The JPHC study consisted of two cohorts. Cohort I recruited participants aged 40 to 59 between1990 and 1994 from five public health centers in different prefectures across Japan (Iwate, Akita, Nagano, Okinawa-Chubu, and Tokyo). Cohort II recruited participants aged 40 to 69 between1993 and 1994 from public health centers in six additional prefectures (Ibaraki, Niigata, Kochi, Nagasaki, Okinawa-Miyako, and Osaka). Baseline surveys were conducted, followed by 5- and 10-year follow-up surveys. The follow-up period extended from 1990 to 2010 for Cohort I and from 1993 to 2013 for Cohort II. The survey (both baseline and follow-up) was composed of a self-administrated questionnaire asking demographic information, height and weight, dietary habits, medical history/condition, and other lifestyle factors. Weight at the age of 20 was asked during the baseline survey (only for Cohort II) and the 10-year follow-up survey (for both Cohort I and II). Body Mass Index (BMI) at the age of 20 was computed using the height reported at baseline. Mortality information, including the cause of death and date of death, was obtained through death certificates collected via public health centers until December 31, 2016. Participants who left their originally registered public health center were considered censored, and their departure dates were confirmed through the residential registry. The cause of death was classified according to the International Classification of Diseases, 10th Revision (ICD10). Out of the 140,420 residents initially recruited for the baseline survey, 277 were deemed ineligible for various reasons (**Supplementary Figure 1**). The response rates at the baseline, 5-year follow-up, and 10-year follow-up surveys were 80.9%, 81.5%, and 87.8%, respectively. Among the 77,171 participants who completed the three surveys, individuals who did not report BMI at any of the four time points (baseline, 5-year follow-up,10-year follow-up, and BMI at the age of 20) (n=8,384), whose BMI were not within a realistic range at any of the four time points (<14 or >40) (n=991), and participants with reported height changes exceeding 10 cm (n=2,194) or reported BMI changes exceeding 10 units (n=82) during the follow-up period were excluded from further analyses. The final sample size for analysis was 65,520. See the flow diagram for detailed information on the exclusion criteria and participant selection (**Supplementary Figure 1**).

**Supplementary Figure S1: Flow diagram of the study.**

**
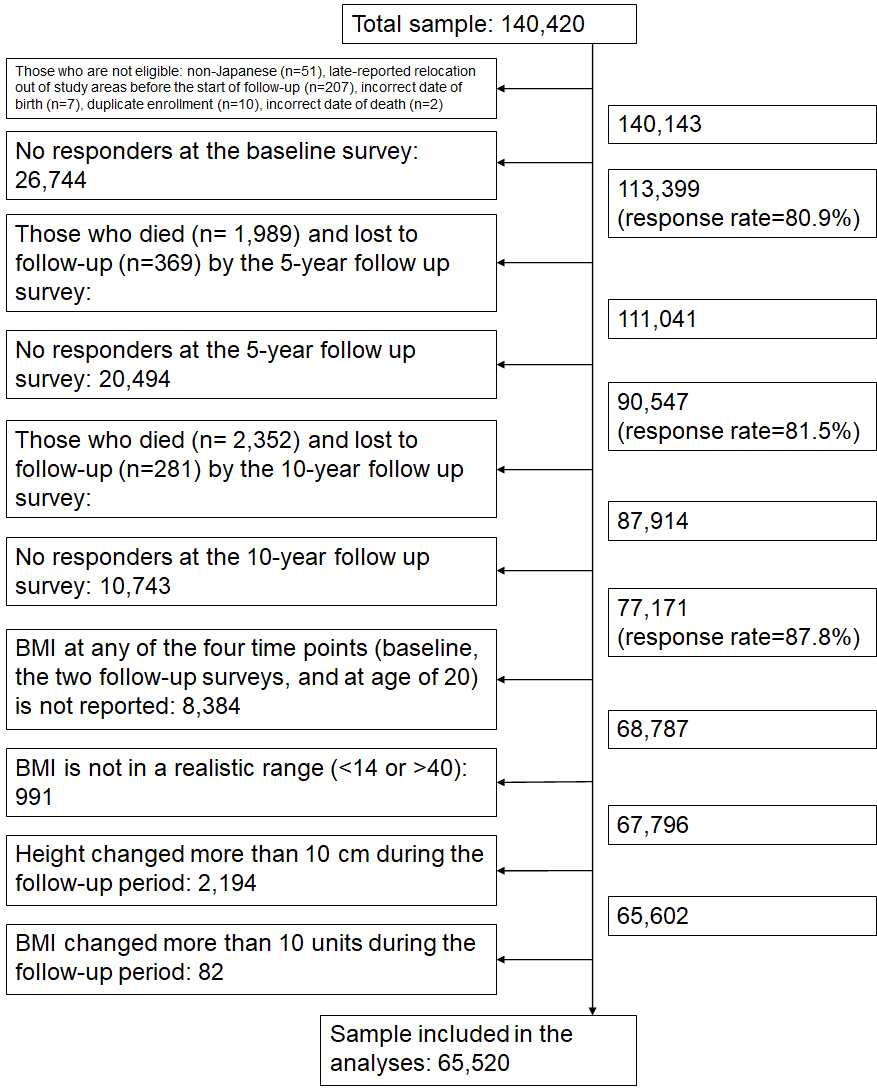
**

BMI (Body Mass Index).

**Supplementary Table S1: Comparison of Akaike Information Criterion, Bayesian Information Criterion, and entropy of different polynomial models**

| **Model** | **Number of parameters** | **AIC**^a^ | **BIC**^b^ | **Entropy** |
| --- | --- | --- | --- | --- |
| **Linear** | 18 | 1134923.75 | 1135087.38 | 0.80 |
| **Quadratic** | 24 | 1127631.41 | 1127849.57 | 0.80 |
| **Cubic** | 30 | 1202365.30 | 1202638.01 | 0.92 |

a: AIC (Akaike Information Criterion). b: BIC (Bayesian Information Criterion).

**Supplementary Table S2: Comparison of Akaike Information Criterion, Bayesian Information Criterion, and entropy of models with different number of groups**

| **Model** | **AIC**^a^ | **BIC**^b^ | **Entropy** |
| --- | --- | --- | --- |
| **1** | 1290907 | 1290962 | 1.00 |
| **2** | 1200357 | 1200467 | 0.80 |
| **3** | 1161266 | 1161430 | 0.82 |
| **4** | 1143583 | 1143801 | 0.82 |
| **5** | 1135775 | 1136048 | 0.79 |
| **6** | 1123030 | 1123358 | 0.81 |
| **7** | 1115603 | 1115985 | 0.81 |
| **8** | 1111006 | 1111442 | 0.78 |

a: AIC (Akaike Information Criterion). b: BIC (Bayesian Information Criterion). *: *P* value < 0.05

**Supplementary Table S3: Estimated Body Mass Index trajectory models for the six groups**

| BMI^a^ trajectory group | Intercept (95%CI^b^) | Age (95%CI^b^) | Age^2^ (95%CI^b^) |
| --- | --- | --- | --- |
| Group 1 | 19.5 (19.4, 19.6)^*^ | 0.040 (0.034, 0.045)^*^ | -0.001 (-0.001, 0.000)^*^ |
| Group 2 | 17.7 (17.6, 17.8)^*^ | 0.173 (0.167, 0.179)^*^ | -0.001 (-0.001, -0.001)^*^ |
| Group 3 | 23.9 (23.7, 24.1)^*^ | 0.013 (0.003, 0.022)^*^ | -0.001 (-0.001, 0.000)^*^ |
| Group 4 | 16.2 (16.1, 16.3)^*^ | 0.327 (0.322, 0.333)^*^ | -0.003 (-0.003, -0.003)^*^ |
| Group 5 | 25.0 (24.6, 25.3)^*^ | 0.125 (0.108, 0.141)^*^ | -0.002 (-0.002, -0.001)^*^ |
| Group 6 | 13.6 (13.2, 13.7)^*^ | 0.569 (0.557, 0.580)^*^ | -0.005 (-0.005, -0.005)^*^ |

a: BMI (Body Mass Index). b: CI (Confidence Interval). *: *P* value < 0.05

1. Tsugane S, Sawada N. The JPHC study: design and some findings on the typical Japanese diet. *Jpn J Clin Oncol*. Sep 2014;44(9):777-82. doi:10.1093/jjco/hyu096
